# Residual flow in false lumen of chronic descending aortic dissection

**DOI:** 10.1007/s12471-017-1052-6

**Published:** 2017-11-09

**Authors:** M. Boulaksil, S. S. Liem, M. Akarkach, J. Timmermans

**Affiliations:** Department of Cardiology, Radboud University Medical Center, Nijmegen, The Netherlands

A 58-year-old male patient has a history of a Stanford type A aortic dissection running up to the left common iliac artery for which a composite aortic valve graft replacement was performed approximately three years ago. This time, the patient presented with fever and chills. We performed a transesophageal echocardiography (TEE), which excluded vegetations.

Upon retracting the TEE probe, the descending aorta showed two compartments (Fig. 1a) separated by a dissected intimal layer (Fig. 1a, arrow heads); the largest compartment is the false lumen (Fig. 1a, hash). Colour Doppler imaging showed laminar flow through the true aortic lumen (Fig. 1a, asterisk). In the false aortic lumen, slow rotating blood flow existed (online video). This phenomenon was not present more proximally in the descending aorta at the aortic arch (Fig. 1b). Residual blood flow may persist in the false lumen years after aortic dissection because of multiple fenestrations in the dissected intimal layer providing entry and exit locations for blood flow. In approximately 70% of patients with acute type A aortic dissection, the dissection extends beyond the ascending aorta [1–3]. After repair, these patients show an increased risk of developing post-dissection aortic aneurysm mainly through false lumen dilatation, requiring late distal aortic re-interventions in up to one-fifth of cases [3, 4]. False lumen patency appeared to be a major risk factor for late re-intervention and was associated with an accelerated annual growth rate [3–5]. Therefore, long-term follow-up and close surveillance of these patients are imperative.

**Fig. 1:**
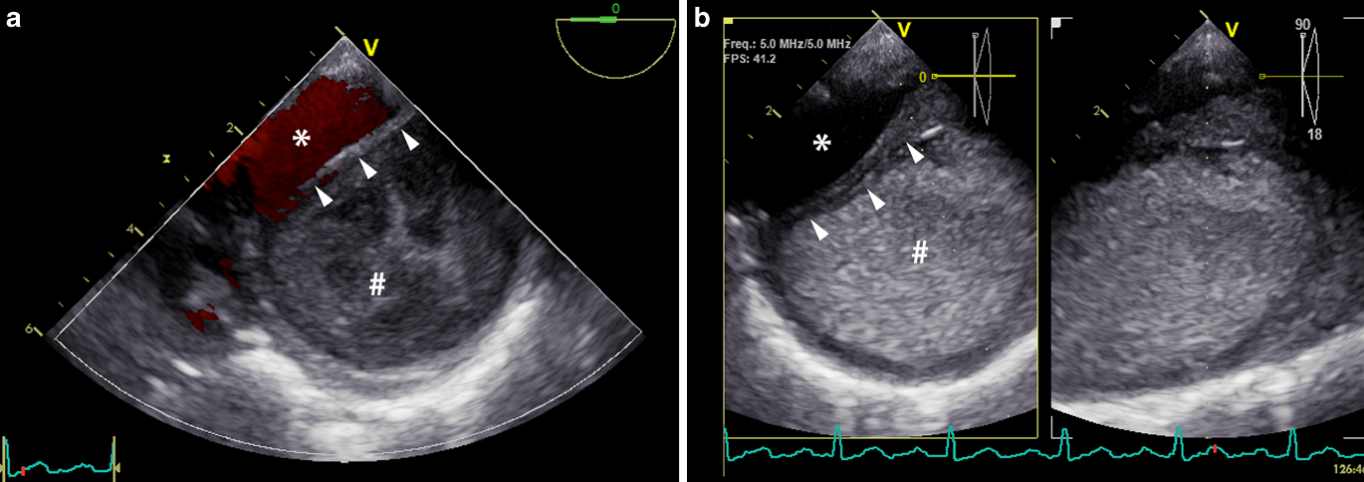
Transverse cross-sectional views of the descending aorta at different levels as documented with TEE. **a** Transversal cross section of the descending aorta showing two compartments separated by a dissected intimal layer (arrow heads). The largest compartment is the false lumen (hash). Colour Doppler showed laminar flow through the true aortic lumen (asterisk) and no entry site to the false lumen. In the false lumen, slow rotating blood flow existed. See also online video. **b** Transversal cross section of the aorta more proximally at the aortic arch. Here, slow rotating blood flow in the false lumen was not observed. *Asterisk* true aortic lumen, *hash* false aortic lumen, *arrow heads* dissection layer

## Caption Electronic Supplementary Material

TEE of transversal cross section of the descending aorta at the same level as depicted in Fig. 1a, showing the true and false aortic lumen. Slow rotating blood flow existed in the false lumen.
